# Whether sports participation is healthy or detrimental for the individual is a matter of dosage

**DOI:** 10.1007/s12471-018-1083-7

**Published:** 2018-02-05

**Authors:** J. L. R. M. Smeets

**Affiliations:** 1 Radboud University Medical Centre, Nijmegen, The Netherlands; 2 Host DAS-CAM, Maastricht University Medical Centre, Maastricht, The Netherlands

That regular strenuous physical exercise is an excellent, effective and ‘cheap’ method in the primary and secondary prevention of cardiovascular disease has been well established. Even in the presence of cardiovascular risk factors and/or cardiovascular disease with or without atrial fibrillation, regular physical exercise improves the health and diminishes the burden of arrhythmias in the individual.

Early studies proving this concept mainly involved the male part of the population, later the positive effect was also confirmed in pre- and post-menopausal females. There is, however, much discussion regarding the optimal level (frequency, duration and intensity) of exercise for maximal beneficial effect. Study results show that the higher the level of aerobic physical activity, the better the decrease in cardiovascular risk profile, in male and female athletes. The aerobic physical activity was expressed in specific time slots per week (7 h per week spread over several days) or METS (metabolic equivalent (MET) is amount of oxygen consumed per kg body weight per minute; in rest +3.5 ml O_2_) to determine the level that would effectively reduce cardiovascular risk, including arrhythmias such as atrial fibrillation [1, 2].

In the last decade, more vigorous exercise has become ‘fashionable’. Marathon runs are very popular and obstacle races in extreme conditions (wet, ice, mud) have gained in popularity.

For 2018, nearly 3,500 races are scheduled worldwide in the ‘ultra’ running category, clearly exceeding the marathon distance. Whether this extreme physical load could cause cardiovascular problems such as atrial fibrillation remains to be determined.

Today, we are confronted with the fact that young athletes, without cardiovascular risk factors or cardiovascular disease are affected by atrial fibrillation, often referred to as ‘lone atrial fibrillation’.

Is there a relation between the level of exercise and the occurrence of atrial fibrillation?

In a cohort study of more than 50,000 long-distance cross-country skiers Andersen et al. found a higher incidence of atrial fibrillation in participants who performed a higher number of races and had faster finishing times. This clearly suggests that a high and prolonged level of exercise is related to the development of atrial fibrillation [3].

Now, endurance sport can be recognised as a cause of cardiovascular disease in specific atrial fibrillation as well [4]. In this issue, Guasch et al. discuss the mechanisms of atrial fibrillation in athletes: what we know and what we do not know [4]. What exactly do we know about the mechanism that initiates and perpetuates atrial fibrillation in an apparently normal heart? Why do some athletes develop atrial fibrillation whereas others performing the same amount of exercise do not? What is the normal behaviour of blood pressure during exercise? Which causative factors might be identified which promote or even cause atrial fibrillation in endurance sport?

Guasch et al. clearly analyse the different components that might be involved in the initiation and maintenance of atrial fibrillation in athletes [4]. Left atrial dilatation is well recognised as a causative factor for atrial fibrillation. Pressure overload, together with some genetic components in the individual, ischaemia and fibrosis formation due to the acute pressure moment with inflammatory reaction can lead to this atrial dilatation and morphologic changes in the atrium and thus to the development of atrial fibrillation.

Atrial premature beats are a classical trigger factor in the triangle of Coumel (substrate, trigger and autonomic nervous system) and play an important role in the initiation and continuation of atrial fibrillation. Bradycardia in the endurance athlete has been attributed to a shift in autonomic balance; a higher parasympathetic tone led to a slow heart rate in rest allowing a larger range in heart rate increase (maximum minus minimum heart rate) to reach maximal exercise. In addition, the shortening of the atrial effective refractory period due to the increase in parasympathetic tone will cause easier initiation of atrial fibrillation. More recently it has been suggested that the intrinsic sinus node function (HCN4 downregulation) might also contribute in severe bradycardia in endurance athletes and thus to the occurrence of atrial fibrillation [5].

Age and sex play a role in the development of atrial fibrillation. Especially the veteran male athletes have a higher chance of atrial fibrillation, which might be related to aging and some form of underlying cardiovascular disease at a yet subclinical level [6]. One of the shortcomings in current research in the field of exercise and atrial fibrillation is the lack of evidence, and studies, concerning the relation between sports activity and atrial fibrillation in female athletes [7, 8].

From this overview, which combines human and experimental animal data, it becomes quite evident that prolonged and strenuous physical exercise may lead to overload of the atrium in such a way that initiation and maintenance of atrial fibrillation is caused. So, dosing the amount of exercise will determine whether sports participation will be beneficial or detrimental for the condition of the heart and thus for the occurrence of arrhythmias such as atrial fibrillation.

Electrical, functional and structural remodelling is important and may be irreversible.

However, an individual approach of the athlete is needed, considering all potential causes for the initiation of atrial fibrillation and avoiding the process leading to irreversible damage and thus to persistence of atrial fibrillation.
